# Longitudinal Brain Functional Connectivity Changes of the Cortical Motor-Related Network in Subcortical Stroke Patients with Acupuncture Treatment

**DOI:** 10.1155/2017/5816263

**Published:** 2017-12-11

**Authors:** Yongxin Li, Ya Wang, Chenxi Liao, Wenhua Huang, Ping Wu

**Affiliations:** ^1^Guangdong Provincial Key Laboratory of Medical Biomechanics, School of Basic Medical Sciences, Southern Medical University, Guangzhou, China; ^2^The 3rd Teaching Hospital, Chengdu University of Traditional Chinese Medicine, Chengdu, Sichuan, China

## Abstract

In clinical practice, the effectiveness of the rehabilitation therapy such as acupuncture combining conventional Western medicine (AG) on stroke people's motor-related brain network and their behaviors has not been systematically studied. In the present study, seventeen adult ischemic patients were collected and divided into two groups: the conventional Western medicine treatment group (CG) and the AG. The neurological deficit scores (NDS) and resting-state functional MRI data were collected before and after treatment. Compared with the CG patients, AG patients exhibited a significant enhancement of the percent changes of NDS from pre- to posttreatment intervention. All patients showed significant changes of functional connectivity (FC) between the pair of cortical motor-related regions. After treatment, both patient groups showed a recovery of brain connectivity to the nearly normal level compared with the controls in these pairs. Moreover, a significant correlation between the percent changes of NDS and the pretreatment FC values of bilateral primary motor cortex (M1) in all patients was found. In conclusion, our results showed that AG therapy can be an effective means for ischemic stroke patients to recover their motor function ability. The FC strengths between bilateral M1 of stroke patients can predict stroke patients' treatment outcome after rehabilitation therapy.

## 1. Introduction

Ischemic stroke is one of the leading causes of morbidity and mortality. Many ischemic stroke patients experience a poor prognosis with neurological and motor function impairment. During the process of improving patients' quality of life, substantial social and economic burden were the cost. Various therapies have been used for poststroke rehabilitation in clinical practice, such as medication, acupuncture, motor training, and surgery [1–5]. Although these treatments can reduce stroke damage and improve patient's behavior, patients are often motor disabled after treatment intervention. So, it is important to identify the biochemical mechanisms underlying stroke-related damage and understand the longitudinal changes of the brain motor network. Rational treatment is needed to help stroke patients' recovery.

Thrombolytic therapies are currently the most effective treatment available after stroke [6]. Recently, acupuncture in addition to thrombolytic medicine is the new treatment therapy for patient after stroke. Acupuncture is a traditional Chinese medicine used to treat many diseases. Some clinical researches have showed that acupuncture is a safe and effective therapy for poststroke rehabilitation and improvement of behavior after ischemic stroke [2, 7]. The mechanism of acupuncture in the functional recovery after stroke has been studied previously [8–10]. A previous study on cerebrovascular responses of stroke patients after acupuncture has found that acupuncture stimulation induced perilesional reorganization through improvement of regional cerebral blood flow [11]. However, no consistent theories were achieved to explore the underlying mechanisms of stroke recovery. The neural mechanism underlying acupuncture treatment after stroke is still unclear.

Neuroimaging, particularly MRI, has become the most powerful tool available to research the underlying mechanisms of stroke and brain functional recovery in stroke patients. The microstructural features and a host of functional characteristics after stroke can be measured by MRI technology. Previous studies based on brain connectivity in stroke patients have revealed that the effects of damage can extend beyond the lesioned area [12, 13]. Our previous diffusion tensor imaging studies have found that the structural integrity of transcallosal motor tracts was damaged after stroke [14, 15]. These previous studies of stroke have proved that MRI technology is a valuable way to detect the effect that the brain acquires after stroke. Moreover, MRI technology has also been used to detect the treatment effect after stroke [10, 16]. Stroke patients with acupuncture treatment showed more improvement in behavioral function and structural integrity than conventional treatment [17]. A previous review study also concludes a similar result that acupuncture treatment improved motor function and the relative fractional anisotropy value at the edge of the ischemic lesions [18]. Most of these previous imaging studies focus on brain organization after stroke. Only a few studies focus on the acupuncture treatment effect, such as the changes in the ipsilesional primary motor cortex (M1) area after stroke with acupuncture intervention [19, 20]. Acupuncture intervention effect on the functional connectivity (FC) patterns of the whole motor-related network is still unclear in stroke patients.

So, the purpose of our present study is to investigate the longitudinal changes in FC of the motor-related network after acupuncture therapy in adult stroke patients. The efficiency of acupuncture therapy comparing with the conventional therapy is also needed to be studied. Based on the previous studies of stroke, we hypothesized that the FC pathways among the cortical motor regions show a recovery for stroke patients after acupuncture therapy. The efficiency of acupuncture therapy in behavioral and FC pattern recovery is higher than that of conventional therapy.

## 2. Materials and Methods

### 2.1. Subjects

Seventeen first-ever stroke patients (8 female, mean age: 63.29 ± 12.27) with unilateral motor deficits due to subcortical ischemic lesions were recruited from the inpatient and outpatient Department of Neurology of the First Affiliated Hospital of the Chengdu University of Traditional Chinese Medicine, China. Some patients had limb dysfunction only, while others had dysfunction in both their upper and lower limbs. Inclusion criteria were as follows: (1) right-handed adults with age ranging from 35 to 80 years; (2) strictly subcortical lesions and absence of other WM pathology as verified by structural MRI; (3) absence of aphasia, neglect, and dementia; (4) no additional neurologic or psychiatric disorders; (5) no previous or subsequent cerebral ischemia; (6) time interval of at least three weeks between stroke onset and the time of study enrollment. Before the enrollment, all patients were only treated with conventional antiplatelet aggregation drugs. None of the patients had undergone any other therapy before the enrollment, such as acupuncture. All of the patients were scanned using resting-state functional magnetic imaging (fMRI) at two time points: before and after one month of clinical treatment. Fourteen healthy control subjects, without neurological or psychiatric disorder and age and gender matched to the stroke group, were enrolled in the study (6 females, mean age: 62.21 ± 10.48). Sample clinical information were provided in Table 1.

This study was carried out in accordance with the recommendations of the Ethics Committee of Chengdu University of Traditional Chinese Medicine with written informed consent from all subjects. All subjects gave written informed consent in accordance with the Declaration of Helsinki. The protocol was approved by the Ethics Committee of Chengdu University of Traditional Chinese Medicine (no. 2011KL-002).

### 2.2. Treatment and Clinical Assessments

After the patients were enrolled, all patients were divided into two groups based on the different treatment protocols used: a continuously conventional treatment of antiplatelet aggregation drug group (CG) or acupuncture combining with conventional drug treatment group (AG). Nine stroke patients were grouped in the CG to give a conventional treatment of antiplatelet aggregation drugs to improve blood circulation (75 mg clopidogrel once each day taken orally, 10 mg *Erigeron breviscapus* injection, and 0.5 g citicoline injection). Eight patients were grouped in the AG to give acupuncture and conventional treatment. Acupuncture was performed at the Baihui (GV-20), Fengchi (GB20, bilateral), Xuanzhong (GB-39, bilateral), Quchi (LI-11 bilateral), Hegu (LI-4, bilateral), Zusanli (ST-36, bilateral), and Sanyinjiao (SP-6, bilateral) acupoints. We selected all of these acupoints because these acupoints are often used in the treatment of motor dysfunction after stroke based on the theory of Chinese medicine. All acupuncture procedures were performed by the time-experienced and licensed acupuncturist: two hours a day for 5 days a week, one week a course, continuous four courses of treatment. The detail operation steps can be seen in our previous study [17]. During the acupuncture treatment process, the dose of medication was adjusted by clinicians according to the patients' conditions.

The clinical performances were assessed before and after treatment to quantify the severity of the neurological functional deficits in the stroke patients using the Chinese Stroke Scale for Clinical Neurological Deficit Scores (NDS, 1995 version). The NDS is an observational test to measure the severity of neurological functional deficit and test the severity of stroke. The NDS consists of 8 items: item 1—awareness (0–9), item 2—horizontal gaze function (0–4), item 3—musculus facialis (0–2), item 4—language (0–6), item 5—upper limb muscle strength (0–6), item 6—manual muscle strength (0–6), item 7—lower limb muscle strength (0–6), and item 8—walking ability (0–6). The resulting score range is from 0 to 45 with lower values reflecting less severity of neurological functional deficit (mild: 0–15 points; moderate: 16–30 points; and severe: 31–45 points). For each group, paired *t*-test analyses were used to detect the changes of DNS before and after treatment. For each patient, percent changes in clinical performance were calculated using the following formula: abs (after − before)/before. These changes were compared between both groups in a two-sample *t*-test analysis.

### 2.3. Image Acquisition

Imaging data were collected using an 8-channel head coil on a 3T Siemens scanner (MAGNETOM Trio Tim, Siemens, Germany) at the West China Hospital MRI Center, Chengdu, China. Resting-state fMRI was collected using an echo-planar imaging sequence with the following scan parameters: TR/TE = 2000/30 ms, FOV = 240 × 240 mm^2^, matrix = 64 × 64, flip angle = 90^0^, slice thickness = 5 mm, 30 interleaved axial slices, and 180 volumes. All participants were instructed to keep their eyes closed and to remain motionless. Foam cushions were used to reduce head translation movement and rotation. All acquisitions were visually inspected for imaging artifacts.

### 2.4. Imaging Processing and Statistical Analysis

We used the seed-based connectivity analysis method to determine the longitudinal FC changes of the motor network in ischemic stroke patients before and after treatment. We computed the connectivity index between each pair of ten region-of-interest areas belonging to the motor execution network [21]. We measured the brain connectivity changes by examining and comparing the brain motor executive network in people recovering from stroke following both interventions and healthy controls from resting-state fMRI. We also tested the correlation between the changes of behavioral performance and the neural imaging index to determine if there are some connections that can be used as a biomarker for evaluating recovery outcomes. The detailed calculation process was performed as follows.

### 2.5. Functional Connectivity Analysis

**Figure 1:**
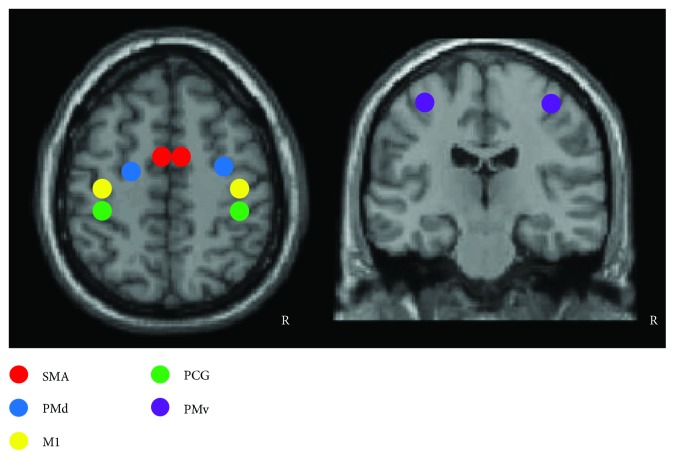
The location of the regions of interest in the motor-related network used in this study. R: right hemisphere.

The resting-state fMRI data was processed using the statistical parametric mapping (SPM8, http://www.fil.ion.ucl.ac.uk/spm) package. Before all of the analyses, images from one patient with a right-sided stroke were oriented around the midsagittal plane prior to data analysis, thereby lateralizing the lesions to the left hemisphere in all patients. The imaging data from the control subject matched to this patient were also midsagittally oriented. The preprocessing steps included slice timing, spatial realignment, normalization into the Montreal Neurological Institute template, and smoothing. A temporal filter (0.01–0.08 Hz) was then applied and nuisance regression was also performed using WM, cerebrospinal fluid, and the six head motion parameters as covariates. Regions of interest (ROIs) for major cortical motor-related areas were selected using ROI-based correlation analysis procedure to assess functional connectivity among the regions. The ROIs included 10 regions, such as bilateral supplementary motor area (SMA), bilateral M1, bilateral postcentral gyrus (PCG), bilateral dorsolateral premotor (PMd), and ventrolateral premotor (PMv). All ROIs were obtained by creating 10 mm-diameter spheres around the predefined coordinates (see Table 2 and Figure 1) [21, 22]. Then, the time series of all voxels in each ROI were extracted and averaged to obtain a mean time series. The between-ROI FC was calculated for each participant. Two-sample *t*-tests between the strokes and the controls were used to detect the differences of the ROI FC (*p* < 0.05, one tailed). In addition, paired *t*-test was also used to detect the longitudinal changes of FC from pre- to posttreatment in both stroke groups. Finally, we tested for relationships between the treatment-related percent changes of NDS and the FC values of each pair ROIs before treatment in all stroke patients. The significance threshold for the correlations was set at *p* < 0.05. To minimize their possible impact on the findings, gender, lesion size, and age were used as covariates of no interest in all the statistical analyses above.

## 3. Results

### 3.1. Behavioral Measures

**Figure 2:**
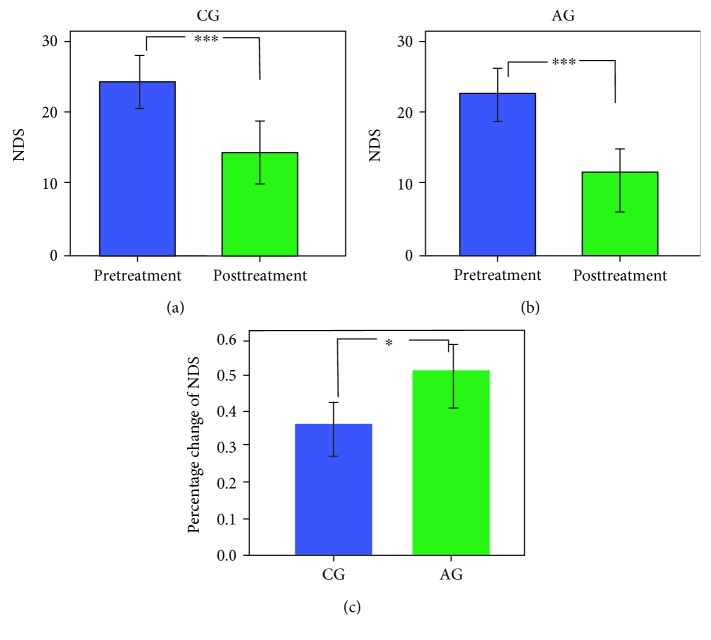
The longitudinal changes of NDS performance with different intervention therapies. Paired *t*-test analyses showed significant decrease of NDS scores from pre- to posttreatment in CG (a) and AG (b). The percent changes of NDS in patients with AG showed a significant enhancement compared with those in patients with CG (c). ^∗^*p* < 0.05 and ^∗∗∗^*p* < 0.001.

Information on all subjects can be found in Table 1. Patients with ischemic stroke exhibited a range from mild to moderate in neurological functional deficit. With treatment intervention, the NDS scores were decreased in all patients. Specifically, more than half of the patients exhibited a mild neurological functional deficit. Paired *t*-test analyses demonstrated that the DNS score was enhanced significantly with treatment intervention for both stroke groups (AG: before 22.86 ± 5.82, after 12.14 ± 4.81, *t* = 15.75, *p* < 0.001; CG: before 23.11 ± 3.79, after 15.44 ± 5.08, *t* = 9.59, *p* < 0.001, see Figures 2(a) and 2(b)). A two-sample *t*-test on the difference in percent changes of NDS between groups showed a significant improvement in AG (AG: 0.495 ± 0.087, CG: 0.355 ± 0.150; *t* = −2.27, *p* = 0.039, see Figure 2(c)).

### 3.2. Altered FC in Stroke Patients Compared with Healthy Controls

**Figure 3:**
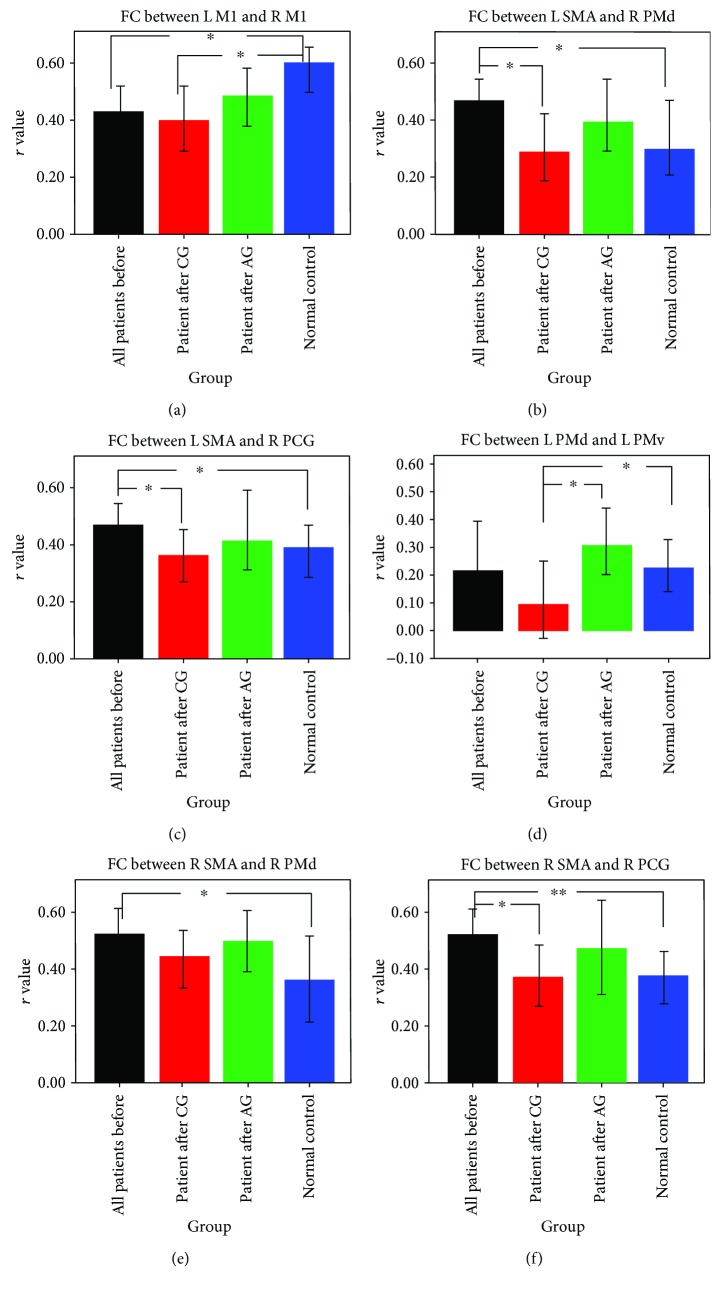
Functional connectivity comparison between groups. Significant interhemisphere connectivity (a–c) and intrahemisphere connectivity (d–f) of the motor network were found for both patients groups comparing with the controls. ^∗^*p* < 0.05 and ^∗∗^*p* < 0.01.

Compared with the controls, stroke patients showed significant changes of interhemispheric FC after stroke: decreased FC between bilateral M1 and increased FC between left SMA and right PMd and PCG (see Figures 3(a), 3(b), and 3(c)). For the patients with CG treatment, abnormal interhemispheric FC in the pair ROIs, such as left SMA versus right PMd and left SMA versus right PCG, was changed to the nearly normal level, but not for the FC between bilateral M1. The connectivity between bilateral M1 after CG still showed a significant decrease comparing with the control group. For the patients with AG treatment, all of this abnormal interhemispheric FC changed to the nearly normal level. Especially for the connectivity pathway between bilateral M1, the FC values were increased after AG treatment (see Figure 3(a)).

In the left hemisphere, intrahemispheric FC between PMd and PMv was increased after AG treatment but decreased after CG treatment (see Figure 3(d)). The FC value of this connectivity pathway showed a significant difference between both patient groups after treatment. The connectivity of other pair ROIs in the left hemisphere did not show significant changes with treatment intervention. In the right hemisphere before treatment, stroke patients showed significantly increased intrahemispheric FC in the pair ROIs, such as right SMA versus right PMd and right SMA versus right PCG (see Figures 3(e) and 3(f)). After treatment, the increased FC between these two pair ROIs in both the CG and AG groups was decreased to the level of no significant compared with the controls.

### 3.3. Correlation between FC Values and Clinical Variable

**Figure 4:**
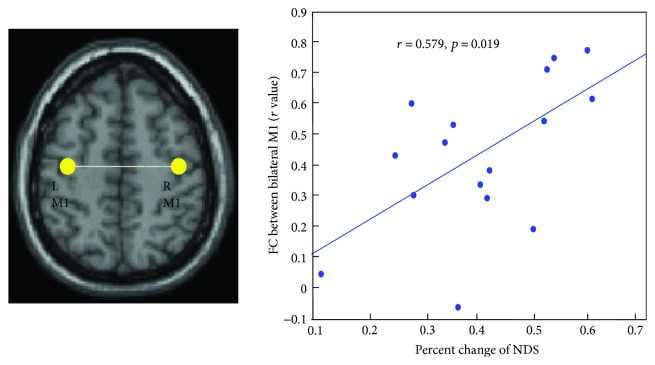
Brain and behavioral correlation. Significant positive correlation was found between the percent changes of NDS and the pretreatment M1-M1 FC values. L: left hemisphere, R: right hemisphere.

Correlation analyses revealed that there was a significant correlation between the percent changes of NDS and the FC values between bilateral M1 before treatment (*r* = 0.579, *p* = 0.019, see Figure 4). No significant correlations were found between the percent changes of NDS and the FC values of other pair ROIs before treatment.

## 4. Discussion

In this study, we examined the treatment effect of acupuncture on stroke patients' motor function through neuroimaging connectivity and clinical behavioral characteristic. Meanwhile, we also explored the relationship between the pretreatment FC values of the motor network and the treatment outcomes in stroke patients. There were several main findings. Firstly, ischemic stroke patients showed a significant change in the FC in the motor network of the stroke patients. With treatment intervention, abnormal FC of the motor network in AG showed more tendency to occur in the nearly normal level than that in CG. Especially, the reduced FC between bilateral M1 was changed to the nearly normal level after acupuncture treatment. Secondly, there was a significant correlation between the percent changes of NDS and the pretreatment FC values between bilateral M1 in all stroke patients. Thirdly, the stroke patients with AG showed a significant decrease in their neurological deficit than those with CG.

### 4.1. Changes of the FC for Patients after Stroke

Considering the altered motor function in stroke patients, an increasing number of studies try to measure the alteration of FC within the motor network (M1, SMA, and lateral premotor cortex) to explore the neural mechanism underlying the disrupted motor function. Previous functional imaging studies have found that stroke caused abnormal interhemispheric FC [21, 23, 24]. Stroke patients showed an increased intrahemispheric FC and a decreased interhemispheric FC [23, 24]. In our present study, the stroke patients showed a significantly decreased FC between the bilateral M1 areas, which is in line with these previous studies. Comparing with the controls, the significant FC changes of the stroke patients in the motor network revealed that the motor network is abnormal after stroke. The brain showed a functional reorganization to adapt the damage after stroke [14]. Previous studies have showed that damages from ischemic stroke result in the functional and structural changes of perilesional and remote brain regions: for example, the structural reorganization of ipsilesional sensorimotor regions and transcallosal and corticospinal connections [15, 25, 26]. So, the exchanges and cooperation between the two sides of the hemisphere were affected by subcortical ischemic stroke. Meanwhile, we also found an increased FC between some pair ROIs of the patients after stroke. The intrahemispheric FC of ROIs in the right hemisphere showed a significant increase in patients after stroke. During our analyses, we have lateralized the lesions to the left hemisphere in all patients. The explanation for the significant increased intrahemispheric FC in the right hemisphere might be a form of compensation for the breaking of brain connectivity balance [27]. Increased FC may be a form of compensation for decreased FC of the contralateral hemisphere brain regions to maintain the motor function of the patients after stroke. Furthermore, our patients were all subcortical stroke patients. The lesions of almost all patients were located in the left hemisphere. Future study should also collect more stroke patients with lesion in the right hemisphere. This can give us complete view of the effect of lesion location on the interhemispheric and ipsilesional disturbances of neural connectivity.

### 4.2. The Treatment Effect of Acupuncture on the FC of the Motor Network

In the present study, our main focus is the treatment effect of acupuncture on the motor function in stroke patients. Two different therapeutic schedules were used to analyze the longitudinal changes with treatment intervention. The significant discovery of our research was that abnormal interhemispheric FC between bilateral M1 was increased to the nearly normal level in the AG but not in the CG. This result verified our hypothesis that FC pathways among the cortical motor regions show a recovery for stroke patients after acupuncture therapy. Acupuncture therapy showed a more significant recovery than only medicine therapy in ischemic stroke. Abundant previous clinical researches have showed that acupuncture was beneficial for stroke rehabilitation [2, 19, 20, 28]. In our experience design, the patients with a combining therapy of acupuncture and drug treatment demonstrated a recovery of the brain FC pattern of the motor network and their neurological function compared with the patients in the CG. Based on the theory of traditional Chinese medicine, these acupoints we used were functionally related to the motor dysfunction after stroke [20, 29]. The needling in these acupoints could modulate the brain function in both hemispheres. A previous imaging study has confirmed that acupuncture can improve regional cerebral blood flow [11]. So, we can say that the acupuncture therapy might accelerate the recovery process of the stroke patients' motor function.

The intrahemisphere FC in the right hemisphere showed a similar treatment effect from both treatment therapies (Figures 3(e) and 3(f)). The results above reveal that both therapies used in our design have a certain effect on the regulation of the motor functional network. However, the treatment effect on the intrahemispheric FC value of the connectivity pathway between left PMd and left PMv showed a significant difference between both patient groups. Acupuncture might modulate regional excitability through influencing the activity of neural synapsis [19, 20]. This effect is more significant in the left hemisphere because the lesions of our data were all located in the left hemisphere. The neural function of the motor cortex was damaged by subcortical ischemic lesions. The experience design by both treatment therapies of the present study can clearly exhibit the treatment effect by acupuncture. Acupuncture might help the recovery process of neural function by improving regional cerebral blood flow [11]. In a behavioral level, the percent changes of NDS in AG showed a significant enhancement compared with the CG. Combining these neuroimaging and behavioral results, we can say that acupuncture has a positive effect on the recovery of motor function. Acupuncture therapy can modulate the interhemispheric and intrahemispheric FC of the motor network in subcortical stroke patients. Future study should also consider the cortical stroke patients. The FC differences between subcortical and cortical stroke should be compared to understand the recovery mechanisms with acupuncture intervention.

### 4.3. Correlation between Connectivity and Behavioral Characteristic

Another significant discovery of our research was the significant correlation between the rate of NDS changes and the pretreatment FC between bilateral M1 in all stroke patients. This correlation between the FC and behavioral scores suggested that the stroke patient with higher FC values between bilateral M1 before treatment showed a better recovery of their neurological functional deficit. Here, we collected all of the stroke patients in this correlation because we want to find predictive biomarkers for future success of rehabilitation therapy for patients after stroke. Both therapies used in our present study showed significant improvement of NDS after intervention. And also, some previous studies on the brain-behavioral correlation were focused on the FC changes and the behavioral changes before and after intervention [30, 31]. In the present study, we wanted to assess whether the preintervention FC can predict behavioral improvement after treatment intervention. A previous review study has implicated that using resting-state activity, one can assess the unique networks for recovery [32]. Using brain connectivity might provide useful guides for therapeutic intervention for patients after stroke. Our present results showed that only pretreatment FC between bilateral M1 showed a significant correlation with the rate of NDS changes in all stroke patients. The role of the interhemispheric M1-M1 connectivity has been investigated by most previous studies [14, 19, 23, 33]. The correlation result in the present study does not only confirm its important role for the FC between bilateral M1 after stroke but also extended its role as a biomarker to predict the treatment outcome. This result would have clinical implication to inform clinical management and choose suitable stroke patients with acupuncture or antiplatelet aggregation drug therapy.

### 4.4. Limitation

Some limitations might affect our results. First, the sample sizes were not large enough. In the future studies, more subjects should be collected to test our results. Second, we only collected the data before and after treatment. The consistent view for the recovery after stroke is that it is a dynamic reorganization. In order to fully understand the neural process of acupuncture treatment effect in stroke, more time points would be needed to collect the neuroimaging data. Thirdly, during the analyses process, we used reversal method to flip the lesions to the left hemisphere for some stroke patients with right lesions. Although this method is usually used in brain lesion studies, this operation can also introduce certain deviations to the analyses. In future studies, larger numbers of patients will be needed to divide the patients into the left- and right-side types of ischemic stroke.

## 5. Conclusion

Changes in FC after treatment suggest brain plasticity in the motor-related network of patients with stroke. The FC strength between bilateral M1 of patients after stroke may be an imaging prognostic marker to predict their recovery outcome of neurological functional deficits. Increased FC in the AG but decreased FC in the CG from pre- to posttreatment in the motor network implicated that acupuncture in addition to Western medicine therapy can be an effective means for ischemic stroke patients to recovery their motor function ability. Our findings provide an imaging basis for the application of acupuncture therapy in clinical treatment.

## Figures and Tables

**Table 1:** Demographic and imaging data.

Patient number	Dominant hand	Affected hand	Site of lesion	Lesion volume (mm^3^)	Lesion age (days)	NDS1	NDS2
AG							
1	R	R	L Pons/CS	350	135	13	5
2	R	R	L/R TH	420	22	31	20
3	R	R	L BG	1180	33	26	12
4	R	R	L BG	3120	21	19	9
5	R	R	L BG/CS	1350	22	26	15
6	R	R	L BG/CS	200	36	—	—
7	R	L	R BG	2240	32	21	10
8	R	R	L TH/LN	1890	148	24	14
CG							
9	R	R	L BG	380	56	24	16
10	R	R	L BG	1190	45	26	23
11	R	R	L BG	540	26	24	18
12	R	R	L BG	1130	132	20	12
13	R	R	L TH	290	21	25	18
14	R	R	L BG	280	23	24	12
15	R	R	L CN	450	25	15	6
16	R	R	L CS	250	23	22	14
17	R	R	L BG	1260	23	—	—

BG: basal ganglia; CN: caudate nucleus; CS: centrum semiovale; LN: lenticular nucleus; F: female; NDS: neurological deficit scores; L: left; M: male; R: right; TH: thalamus.

**Table 2:** Regions of interest for the major cortical motor-related areas.

ID	Regions	Abbreviation	Side	MNI coordinate		
				*X*	*Y*	*Z*
1	Supplementary motor area	SMA	L	−5	−4	57
2	Supplementary motor area	SMA	R	5	−4	57
3	Primary motor cortex	M1	L	−38	−22	56
4	Primary motor cortex	M1	R	38	−22	56
5	Postcentral gyrus	PCG	L	−37	−34	53
6	Postcentral gyrus	PCG	R	37	−34	53
7	Dorsolateral Premotor	PMd	L	−22	−13	57
8	Dorsolateral Premotor	PMd	R	28	−10	54
9	Ventrolateral Premotor	PMv	L	−49	−1	38
10	Ventrolateral Premotor	PMv	R	53	0	25

The regions were selected from previous studies (Jiang et al. [22] and Wang et al. [21]). The location of each region of interest with a 5-radius sphere. L: left; R: right; MNI: Montreal Neurological Institute.
